# The bioavailability and blood levels of low-dose rapamycin for longevity in real-world cohorts of normative aging individuals

**DOI:** 10.1007/s11357-025-01532-w

**Published:** 2025-01-28

**Authors:** Girish Harinath, Virginia Lee, Andy Nyquist, Mauricio Moel, Maartje Wouters, Jesper Hagemeier, Brandon Verkennes, Colleen Tacubao, Sayem Nasher, Krister Kauppi, Stefanie L. Morgan, Anar Isman, Sajad Zalzala

**Affiliations:** 1 AgelessRx, Ann Arbor, MI USA; 2 Division of Research and Applied Sciences, AgelessRx, Ann Arbor, MI USA; 3 Data and Analytics Division, AgelessRx, Ann Arbor, MI USA; 4 Rapamycin Longevity Lab, Gothenburg, Västra Götaland County Sweden

**Keywords:** Rapamycin, Bioavailability, Compounded, Commercial, Longevity, Clinical study

## Abstract

Rapamycin, also known as sirolimus, has demonstrated great potential for application in longevity medicine. However, the dynamics of low-dose rapamycin bioavailability, and any differences in bioavailability for different formulations (e.g., compounded or commercial), remain poorly understood. We thus explored rapamycin bioavailability in two real-world cohorts to begin providing a foundational understanding of differences in effects between formulations over time. The small trial study cohort was utilized to explore the blood rapamycin levels of commercial (*n* = 44, dosages 2, 3, 6, or 8 mg) or compounded (*n* = 23, dosages 5, 10, or 15 mg) rapamycin 24 h after dose self-administration. Results suggested dose-to-blood level relationships were linear for both formulations, though compounded had a lower bioavailability per milligram of rapamycin (estimated to be 31.03% of the same dose of commercial). While substantial inter-individual heterogeneity in blood rapamycin levels was observed for both formulations, repeat tests for individuals over time demonstrated relative consistency. Extending exploration to 316 real-world longevity rapamycin users from the AgelessRx Observational Research Database produced similar findings, and additionally suggested that blood rapamycin levels peak after 2 days with gradual decline thereafter. Taken together, our findings suggest that individualized dosing and routine monitoring of blood rapamycin levels should be utilized to ensure optimal dosing and efficacy for healthy longevity.

**Supplementary Information:**

The online version contains supplementary material available at 10.1007/s11357-025-01532-w.

## Introduction

The number of people aged > 65 years is expected to reach 2 billion by 2050, which will present significant challenges given the evidence that biological aging is the biggest risk factor for age-related chronic diseases such as cancer, cardiovascular disease, and neurodegenerative diseases [1–3]. The high comorbidity of age-related diseases in elderly individuals limits the benefit that can be obtained by targeting each chronic disease individually. As such, it is essential to implement preventative healthcare strategies that address the biology of aging to limit the rise of multi-morbidity [4, 5].

The translational geroscience field has placed substantial focus on assessing gerotherapeutics targeting the molecular mechanisms underlying biological aging, with the aim of improving healthy longevity [6]. In the past 15 years, several gerotherapeutics have shown efficacy in improving healthy lifespan in preclinical models; however, whether these therapeutics can delay or even prevent multiple age-related diseases in humans remains poorly understood. To address these gaps, translational geroscientists are evaluating US Food and Drug Administration (FDA)-approved interventions for their potential to mitigate age-related decline and be repurposed as gerotherapeutic interventions. Among these, the small molecule rapamycin has been most broadly recognized to hold significant translational promise [7–9].

Rapamycin inhibits the mechanistic target of rapamycin (mTOR), a serine/threonine kinase composed of two functionally distinct complexes, mTOR complex 1 (mTORC1) and 2 (mTORC2). The activities and substrate specificities of mTORC1/2 are regulated by complex co-factors to collectively serve as a molecular control for the maintenance of cellular homeostasis [10]. Potent chemical inhibition of mTORC1 by rapamycin has been extensively characterized and has demonstrated considerable efficacy in preclinical studies for addressing age-related diseases such as cancer, cardiovascular disease, neurodegenerative disease, and sarcopenia [11–13]. This has spurred significant interest in rapamycin and its derivatives as gerotherapeutics, and early studies in companion animals, non-human primates, and small cohorts of aged individuals have shown promising results [14, 15]. However, further investigation in longitudinal, randomized controlled trials (RCTs) in large and diverse normative aging cohorts is necessary to obtain a more comprehensive understanding of rapamycin’s potential for longevity medicine.

Several clinical trials assessing the effects of various dosing and regimen schedules of rapamycin on age-related decline are currently underway in healthy adults (such as the PEARL trial NCT04488601) [14]. These are of importance to the geroscience field, as existing data on dosing regimens that extend lifespan have only come from model organisms. Thus, our current knowledge cannot be directly translated to human doses, as factors such as interspecies differences in bioavailability, half-life, clearance, plasma protein binding, and tissue distribution play critical roles in functional outcomes [16]. As such, understanding these factors in normative aging individuals will be essential for the effective application of rapamycin as a gerotherapeutic in the future.

For FDA-approved uses of rapamycin, such as anti-rejection for kidney transplants, and treatment of tuberous sclerosis, solid tumors, and lymphomas, the pharmacokinetics (PK) have been thoroughly characterized [17–20]. However, this information is likely not applicable to the use of rapamycin for promoting healthy aging and longevity, as patients receiving rapamycin for FDA-approved conditions have compromised physiological states and may be taking multiple other drugs, which may not effectively translate to gerotherapeutic use in healthy individuals [9, 14, 21, 22]. This is an important consideration as data on the clinical application of rapamycin for healthy aging is still in a nascent state and requires a nuanced approach. Specifically, it must prioritize avoiding adverse events (AEs) to achieve a geroprotective therapeutic dose range while simultaneously minimizing side effects [23–25].

A small but impactful body of work from Joan Mannick’s lab has provided evidence of safety and tolerability of low-dose everolimus (5 mg per week) and further demonstrates improvements for the aging immune system in a cohort of elderly humans [26, 27]. While this provided initial baseline parameters on dosing and dosing schedules for gerotherapeutic applications of rapamycin, many questions remain. Of particular importance, data on the bioavailability and blood rapamycin dynamics in normative aging individuals following weekly administration of low-dose rapamycin (purported “longevity doses”) is relatively non-existent. Despite these unknowns, recent estimates suggest that thousands of people across the USA are currently taking rapamycin off-label at various longevity dosages [26, 28]. Indeed, we recently described real-world data from a cohort of 333 participants using off-label rapamycin across a wide range of dosages, regimens, and formulations [29]. This study suggested rapamycin can be used safely in normative aging adults over extended periods of time with over one-third of rapamycin users self-reporting benefits in mood, pain, cognition, and fewer moderate to severe acute coronavirus disease 2019 (COVID-19) cases than non-users over the study time period. Consistent with a majority of anecdotal reports, 6 mg/week rapamycin was by far the most commonly administered longevity dose. While this initial evidence is promising, effective validation of proclaimed longevity doses of rapamycin requires a deeper exploration of the bioavailability and therapeutic blood levels that must be achieved in normative aging individuals for therapeutic effects, especially if there is significant inter-individual variability in response to rapamycin.

Individuals using rapamycin off-label as a gerotherapeutic are increasingly turning to its compounded forms, which provide more precise dose tailoring, higher dose capsules for easier administration and adherence, easier placebo capsule design for RCTs, and greater affordability [30, 31]. Although rapamycin is categorized as a small molecule, it is relatively large for its class and is lipophilic, resulting in a low solubility in the digestive tract, which may compromise its absorption and bioavailability [20, 32, 33]. This has resulted in broader concerns about the bioavailability of compounded rapamycin [34, 35]. Additionally, individual characteristics (e.g., sex, body mass index (BMI), diet, and genetic polymorphisms) have been shown to influence the bioavailability of rapamycin, but are not yet well understood [36–39]. Further, differing methodologies and quality control practices between compounding pharmacies may result in meaningfully different products [40]. Taken together, skepticism surrounding the use and bioavailability of compounded rapamycin remains, despite its potential advantages.

To begin to address this knowledge gap, we conducted a study with two real-world cohorts to explore the relative bioavailability of commercial and compounded rapamycin. We collected measurements of 24-h blood rapamycin levels in 67 individuals stratified by dosage, sex, and BMI, and compared these findings with retrospective results from 316 individuals in our Observational Research Database with blood level measurements of rapamycin spanning 7 days post-administration. We further evaluated inter- and intra-individual heterogeneity in bioavailability in individuals taking the same dose of rapamycin across different time points, as well as the stability of blood rapamycin levels in individuals taking rapamycin over 6–18 months. These efforts aim to contribute to the development of more effective rapamycin dosing protocols for healthy aging, and to inform the design of more rigorous RCTs aimed at validating rapamycin as a putative gerotherapeutic.

## Methods

### Study design

This study collected real-world data to better understand the bioavailability of compounded and commercial rapamycin, with a specific focus on observing patterns of rapamycin levels in the blood (blood rapamycin levels) following longevity doses of rapamycin. It was conducted in two parts—a small trial study cohort that opted into sirolimus testing following an explicit set of directions for a dose-blood test protocol, and a retrospective cohort of AgelessRx’s Observational Research Database participants, who agreed to share basic, de-identified information about their longevity medicine journey, including rapamycin blood levels, for future research purposes. The study protocol was approved by the Institutional Review Board of the Institute of Regenerative and Cellular Medicine (IRCM, approval number IRCM-2022–352), and was conducted in accordance with Good Clinical Practice (GCP) standards.

### Participants

All participants were healthy, active AgelessRx patients receiving rapamycin treatment (compounded or commercial) for its potential longevity and health improvement benefits. Participants who sought rapamycin prescription for longevity support, pain reduction, energy improvement, weight loss, mood improvement, sleep improvement, or oral health improvement were included in the study. To be eligible to receive AgelessRx’s standard rapamycin treatment protocol, participants had to be ≥ 40 years of age, without a history of uncontrolled disease.

The trial study cohort included 67 participants (24 females and 43 males) who responded to email-based recruitment outreach to participate in blood testing for rapamycin levels. The larger Observational Research Database cohort included all AgelessRx users taking rapamycin for longevity who had opted in to sharing data with the research team for anonymized research purposes, had blood sirolimus level tests available, and who had reported both the day of the week they had taken their most recent dose of rapamycin, as well as when they had completed bloodwork for sirolimus levels thereafter. This resulted in data from 316 individuals (87 females, 229 males) being available for inclusion in this study. Only the data points necessary to address questions directly relevant to this study were extracted from participant files for analysis in this work.

### Treatment

Those requesting rapamycin and deemed eligible for prescription by the medical team were prescribed compounded or commercial rapamycin, based on personal preference, for both portions of the study. The trial study cohort included 23 participants prescribed compounded rapamycin at dosages 5 mg, 10 mg, or 15 mg (beginning at 5 mg/week and titrating up to 15 mg/week, if tolerated without AEs), and 44 participants prescribed commercial rapamycin at dosages of 2 mg, 4 mg, 6 mg, or 8 mg (with titration beginning at 2 mg/week and titrated up to a target dose of 6 mg/week as needed in 2 mg increments, with the option to increase to 8 mg/week if requested). The Observational Research Database cohort included primarily commercial rapamycin users at 2–8 mg per week with a high representation of 6 mg (~ 80%), though 12 individuals using compounded rapamycin (10 mg) were included. For both cohorts, the commercial (tablets; Dr. Reddy’s Laboratories Limited, Princeton, NJ, USA) and compounded (tablets) rapamycin were dispensed and distributed to participants by Precision Compounding Pharmacy (Bellmore, NY, USA), which provided certifications of validation for these products. Of important note, as compounded rapamycin is more prone to impurities and inefficacy if vendors do not have adequate quality control and assurance certifications to ensure purity, the compounded rapamycin utilized in our study came from a single pharmacy to limit variability caused by different compounding pharmacy protocols, standards, and practices. It was carefully vetted for rigorous quality control, and is both NABP accredited and LegitScript certified. Nonetheless, independent validation of the potency of the compounded and generic rapamycins used in this study, conducted by NEXT Molecular Analytics (Chester, VA) using HPLC analysis, suggested that the compounded rapamycin doses contained an average of 26% less rapamycin per mg than the generic formulation. While this discrepancy could partly be explained by technical challenges in the validation method used (e.g., differences in extraction efficiency due to the different inactive ingredients in each formulation), we cannot eliminate the possibility that the compounded rapamycin is less potent per milligram at baseline. Taken together, we stress that careful evaluation of compounded rapamycin sources and ongoing monitoring of materials supplied is critical for appropriate use.

Dosing advice for all groups was to take their formulation of rapamycin on the same day of each week, any time of day, with or without meals. For all participants, the dose taken by an individual was largely determined by personal preference in conjunction with provider suggestions to maximize efficacy for managing symptoms of aging (e.g., if the individual was looking to reduce pain symptoms and was achieving some success, but sought more, they might increase the dose if well tolerated), while aiming to simultaneously limit any undesirable side effects.

### Assessments

Participants in the trial study were sent surveys to complete. The survey asked three questions on rapamycin dosage administered, date and time of administration, and any additional information they wished to share with the AgelessRx research team, in which they were prompted to provide open-ended responses. Participants were instructed to go to the nearest Quest Diagnostics Laboratory location for a blood draw as close to 24 h after rapamycin administration as possible to measure blood sirolimus (commercial name of rapamycin) levels. The research team discussed Quest lab locations with each participant before the start of their rapamycin protocol. A requisition for the “Sirolimus assay” (Test code 36,712; Quest Diagnostics) was provided to Quest Diagnostics in advance. This test is designed to measure blood sirolimus/rapamycin levels by liquid chromatography/tandem mass spectrometry. Quest Diagnostics reported the collection date and time with the results of the Sirolimus assay to AgelessRx. The time and date were compared with the participant-completed survey to determine the time between dosage and blood collection. Any participants who did not have a Quest Diagnostics laboratory within reasonable proximity were directed to alternate facilities and followed the same procedure.

As the option to participate was sent each time a participant expressed interest in measuring blood rapamycin levels, several interested participants completed additional blood draws to evaluate their Sirolimus assay values while taking the same or higher doses of rapamycin, based on their titration schedule. Each dose of rapamycin was spaced apart by one week. Twenty-one participants provided multiple blood draws, which included 15 participants in the compounded group (12 using a different dose, and 3 with the same dose) and 6 participants in the commercial group (1 with a different dose, and 5 with the same dose) (Supplementary Table S5). Cross-sectional analyses were performed using the value obtained after the most recent dose and Sirolimus assay reading (unless otherwise described). We chose this methodology with the assumption that the most recent dose was the dosage determined to be the optimal maintenance dose for the participant and captured the highest doses of rapamycin taken, which is of most clinically relevant interest. Further, as most individuals were taking rapamycin for different periods of time, assessing the most recent dose helped ensure we were uniformly collecting data from individuals taking rapamycin for longer periods of time. For the purposes of this report, we will use the wording blood rapamycin levels when referring to the Sirolimus assay results.

### Statistical analysis

Normality was tested using the Shapiro–Wilk test and visual inspection of Q-Q plots. Commercial and compounded groups were compared using Student’s *t*-test or Welch’s *t*-test in case of unequal variances for continuous measures, and Chi-squared for categorical measures. Due to the constraints of the real-world retrospective study, data on participants’ baseline levels of rapamycin prior to taking their rapamycin dose was not always available, and as such was not included in this study. As appropriate, linear regression or linear mixed-effect models with random intercept per subject were fitted to test for the main effect of protocol (compounded vs. commercial) and the interaction effect of protocol by dosage. The models included repeated measures as a within-subject factor and were adjusted for age, sex, and BMI. Statistical analysis was conducted using SPSS 28 (IBM Corp, Armonk, NY, USA), and Python 3.8 using the SciPy and matplotlib packages for data visualization. *P*-values < 0.05 were considered statistically significant.

## Results

**Fig. 1:**
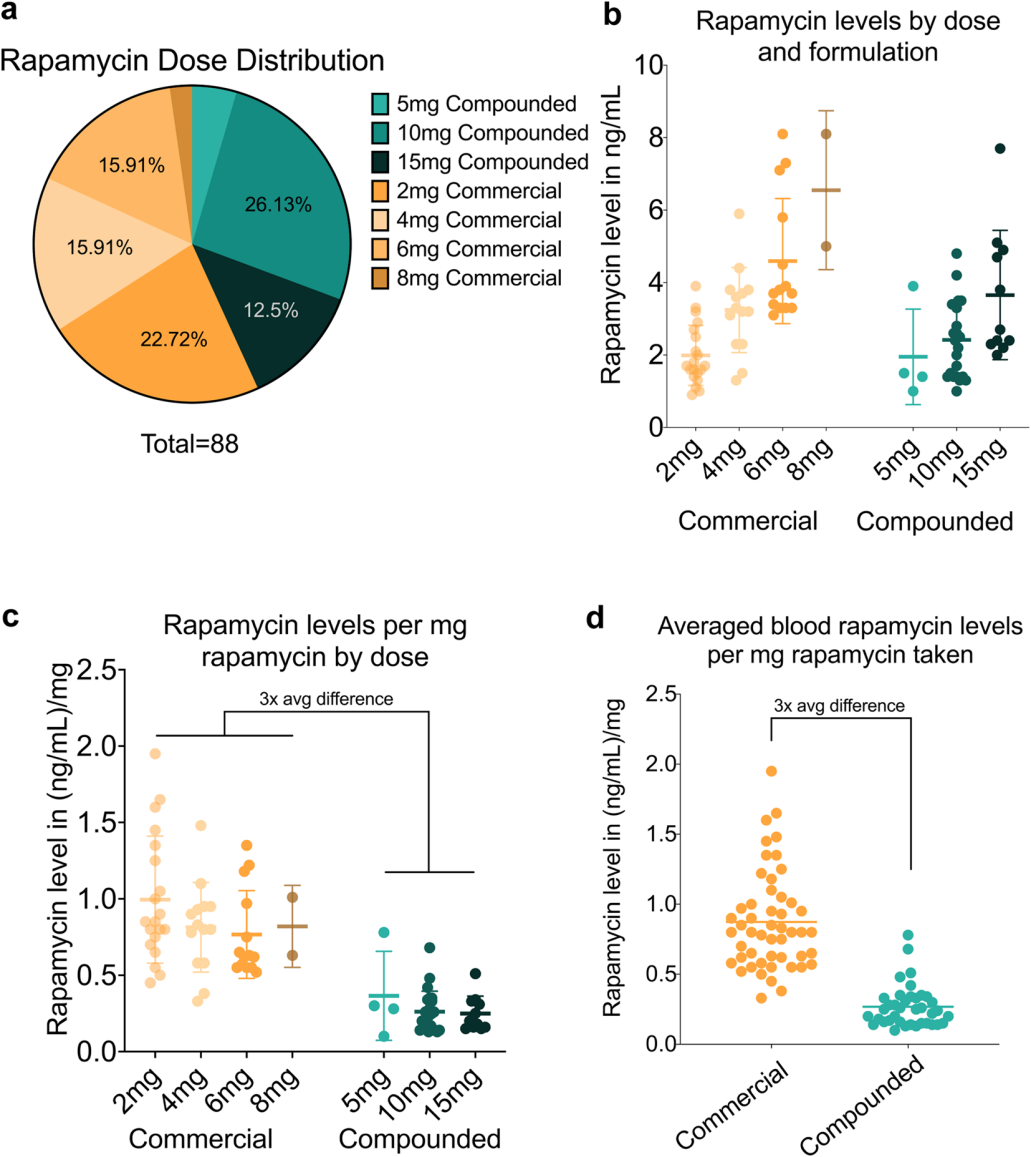
Impact of rapamycin formulation and dose on bioavailability. Quantities of rapamycin (in mg) taken by participants encompassed a moderate span of the gerotherapeutic dosing range (**a**). Rapamycin concentration in the blood was relatively similar for compounded (mean = 2.73, SD = 1.4) and commercial (mean = 3.25, SD = 1.8) formulations when stratifying by dose (**b**), however, normalizing blood rapamycin levels to the amount of rapamycin taken for each dose (**c**) and averaging across doses to obtain a final estimate revealed compounded rapamycin (mean = 0.27 ng/mL per 1 mg) is 31.03% (3 ×) less bioavailable per milligram than commercial formulations (mean = 0.87 ng/mL per 1 mg) (**d**). Bars represent confidence interval of the mean (**b**) or standard deviation (**c**), and lines represent mean

The 67 participants recruited to participate in the small trial study of rapamycin bioavailability included 24 females and 43 males who were routinely taking either compounded (*n* = 23 at 5, 10, or 15 mg) or commercial rapamycin (*n* = 44 at 2, 4, 6, or 8 mg), with the specific dose per individual being determined by a partnership between patient and physician to optimize the balance between healthy aging benefits and side effect tolerance. While age was statistically higher for individuals taking compounded rapamycin (compounded mean = 61.7 years, SD = 9.1; commercial mean = 56.8 years, SD = 9.3; t(65) =  − 2.050, *p* = 0.044), and gender distributions had a non-significantly higher number of males than females in both groups (compounded: males = 17 (74%), females = 6 (26%); commercial: males = 26 (59%), females = 18 (41%); $\chi$ ^**2**^ (1, *N* = 67) = 1.443, *p* = 0.230), BMI was similar across both groups (compounded mean = 23.8 kg/m^2^, SD = 2.9; commercial mean = 25.1 kg/m^2^, SD = 4.8; t(65) = 1.158, *p* = 0.251; Supplementary Table S1, Supplementary Figure S1). Participant distribution across doses is summarized in Supplementary Table S2 (Fig. 1a).

For all rapamycin formulations and doses, blood rapamycin levels were evaluated 24 h after dosing. Stratifying blood rapamycin levels by dose suggested that levels across the dosing ranges used for each formulation were somewhat similar (Fig. 1b), though clearly not equivalent, by dose. This was not fully explained by differences in potency of the two formulations (compounded was independently validated to be approximately 75% as potent as commercial per milligram), and no significant association was observed between blood rapamycin levels and BMI, gender, length of time taking rapamycin, self-reported activity level, pre-existing health conditions, or other medications taken simultaneously for either formulation (see Supplementary Table S3 for a summary). However, given the differences in blood rapamycin levels per dose for each formulation, we evaluated the average rapamycin blood level per mg of rapamycin taken to better understand differences in bioavailability for each formulation (Fig. 1c, Table 1). To obtain an estimate of comparative efficacy, the per mg bioavailability for each formulation was examined both as an average for each dose as well as an average across all doses (Fig. 1d, Table 1). From this, we estimated blood rapamycin levels of compounded to be 0.27 ng/mL per 1 mg dose, and commercial to be 0.87 ng/mL per 1 mg dose, resulting in a 31.03% bioavailability of compounded rapamycin relative to commercial rapamycin.

**Table 1:** Bioavailability of all rapamycin datapoints by dose and formulation

	*N*	Mean rapamycin blood levels (SD) in ng/mL	Mean rapamycin blood levels (SD) in (ng/mL)/mg
Compounded	38	2.73 (1.4)	0.27 (0.1)
5 mg	4	1.95 (1.3)	0.37 (0.3)
10 mg	23	2.42 (1.1)	0.26 (0.1)
15 mg	11	3.66 (1.6)	0.25 (0.1)
Commercial	50	3.25 (1.8)	0.87 (0.4)
2 mg	20	1.99 (0.8)	1.00 (0.4)
4 mg	14	3.24 (1.8)	0.81 (0.3)
6 mg	14	4.59 (1.7)	0.77 (0.3)
8 mg	2	6.55 (2.2)	0.61 (0.4)

*SD* standard deviation

**Fig. 2:**
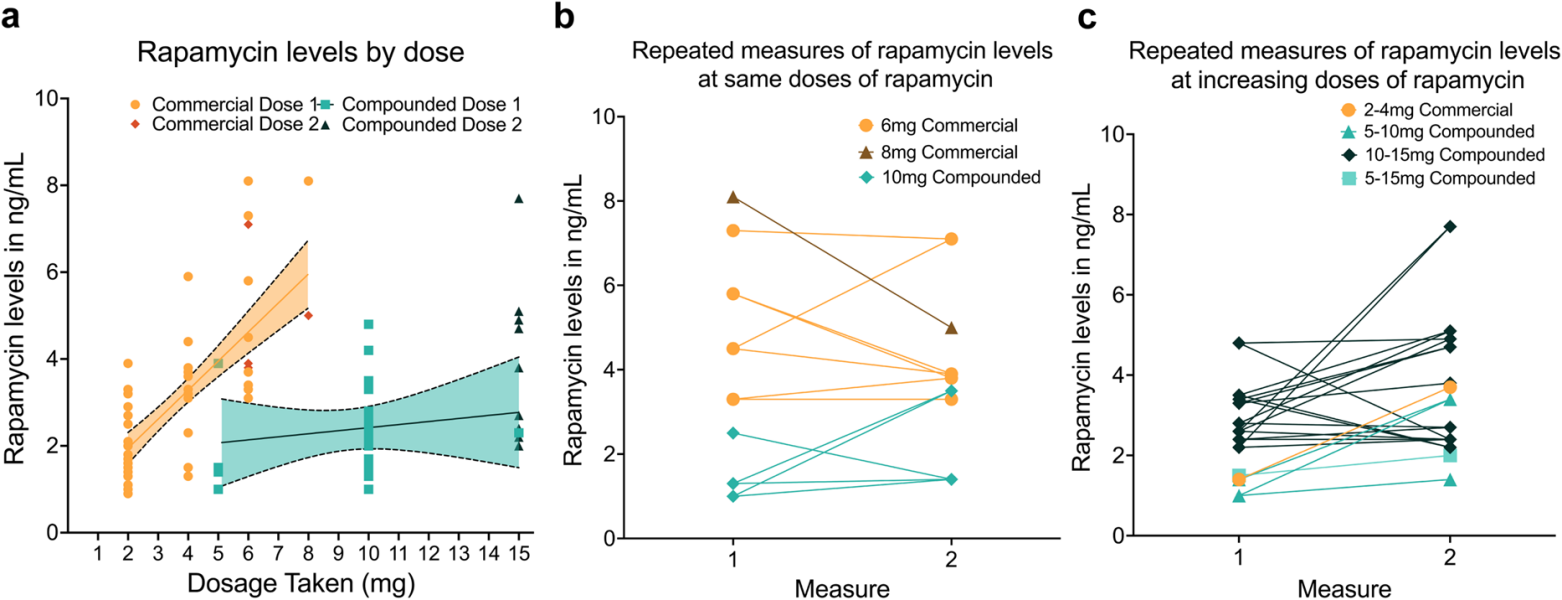
Heterogeneity of dose-to-blood level bioavailability. Compounded and commercial formulation groups both demonstrated significant association between dose and blood level (compounded B = 0.173, SE = 0.071, t(25.6) = 2.442, *p* = 0.022; 95% CI = 0.027–0.319, commercial B = .697, SE = 0.111, t(38.5) = 6.269, *p* < 0.001, 95% CI = 0.472–0.922), with a significant interaction effect (B: − 0.524; SE: 0.130, t(78.6) =  − 4.026, *p* < 0.001; 95% CI =  − 0.783 to − 0.265) suggesting significantly different increases in bioavailability by formulation across doses (**a**). Despite high inter-individual heterogeneity in bioavailability at a given dose of rapamycin, repeated doses in the same individuals showed consistency in bioavailability (**b**), and increasing doses in the same individuals tended to increase blood rapamycin levels (**c**), with no meaningful differences between formulations. CI, confidence interval; SE, standard error

We next explored the linearity of rapamycin dose to blood concentration for the study cohort using mixed effect models. Within both compounded (B = 0.173, SE = 0.071, t(25.6) = 2.442, *p* = 0.022; 95% CI = 0.027–0.318) and commercial (B = 0.697, SE = 0.111, t(38.5) = 6.269, *p* < 0.001, 95% CI = 0.472–0.922) groups, significant associations were observed between dose and blood rapamycin levels (Fig. 2a, Supplementary Table S4.1), though the slope of the dose-to-blood curve differed significantly between the two formulations, (B =  − 0.524; SE = 0.130, t(78.6) =  − 4.026, *p* < 0.001; 95% CI =  − 0.783 to − 0.265; Fig. 2a, Supplementary Table S4.2). This suggests that while both formulations had linear dose-to-blood level relationships, commercial rapamycin elicits a significantly stronger response in blood rapamycin levels as dosage is increased compared to compounded.

Importantly, however, we noted substantial inter-individual variability in blood rapamycin level concentrations at each dose of compounded and commercial rapamycin administered, suggesting differences in bioavailability between people taking the same rapamycin dose independent of formulation (Fig. 2a). Thus, to more fully evaluate whether this variability is inherently characteristic of rapamycin overall or is rather a facet of an individual response, we explored blood rapamycin levels in participants for whom two measurements were available within 30 days (compounded *n* = 15, 12 with increasing doses and 3 with same doses; commercial *n* = 6, 1 with increasing doses and 5 with the same doses; Supplementary Table S5). For those taking the same dose of rapamycin at both timepoints (compounded, *n* = 3; commercial, *n* = 5), blood rapamycin levels were similar between the first and second measurement of the same dose for most (though not all) participants, suggesting a relatively stable individual response (Fig. 2b). Similarly, for participants receiving two differing doses, the second (higher) dose tended to be followed by an expected higher blood concentration of rapamycin, though some variability in response was again observed (Fig. 2c). No meaningful differences were observed in trends between compounded and commercial formulations.

**Fig. 3:**
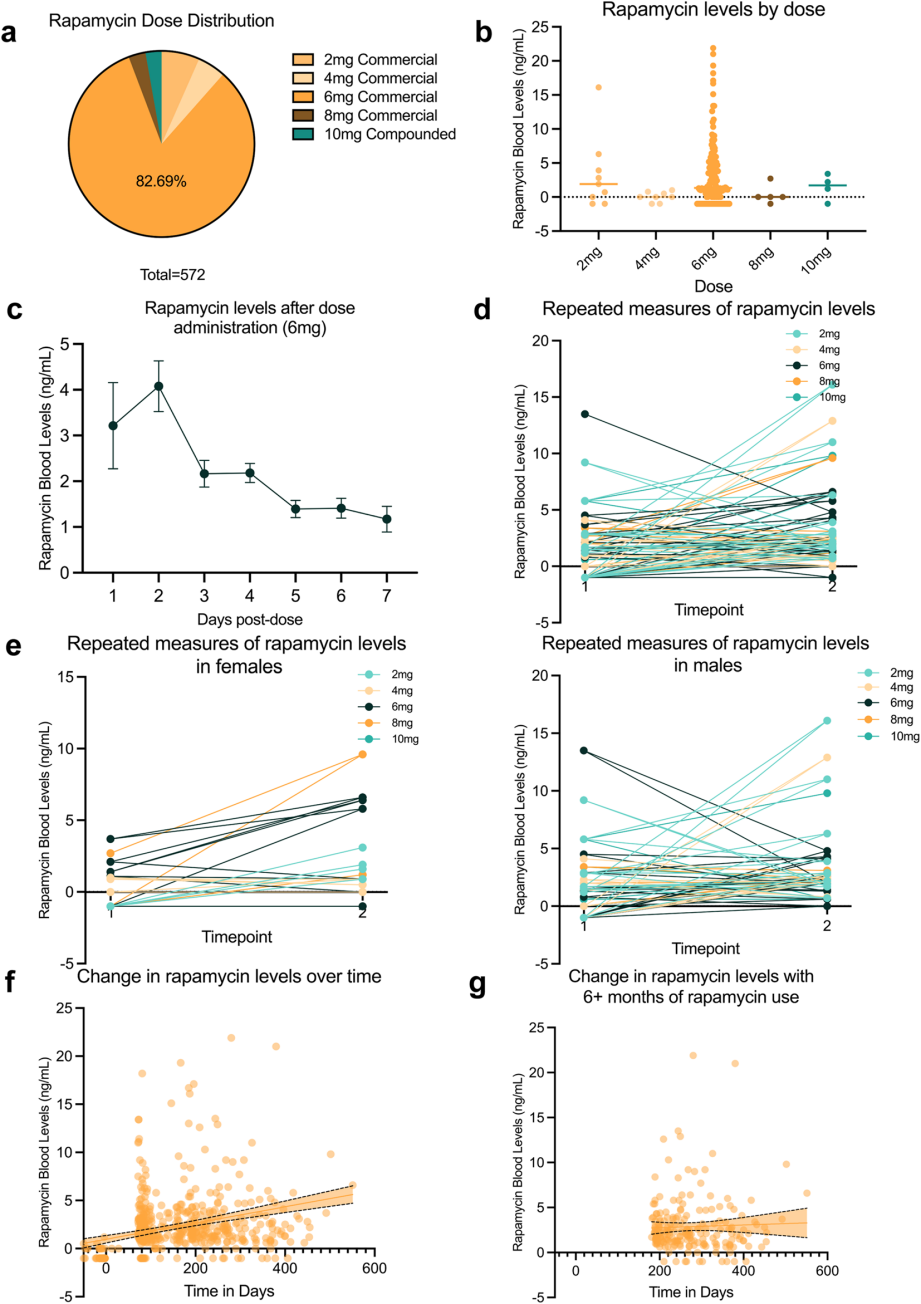
Real-world rapamycin user bioavailability over time. Blood rapamycin levels from 316 real-world rapamycin for longevity users (**a**), 167 of whom had dose-test intervals of 48 h or less, demonstrated consistent distribution of bioavailability for each represented dose of commercial and compounded rapamycin formulations (**b**). Averages of blood rapamycin levels for individuals taking 6 mg rapamycin spanned 7 days post dosing, and showed the highest levels at 2 days post dosing (**c**). For individuals with repeat measurements across two timepoints, blood rapamycin levels tended to increase overall (**d**), in both sexes (**e**). Trends of improvement over time were seen when evaluating all datapoints over the entirety of the available range (**f**), but showed stabilization when restricted to those who had been using rapamycin for 6 months or more (**g**)

Given the inherent limitations of small datasets, we expanded our investigation on rapamycin bioavailability dynamics to our Observational Research Database, which collects real-world data from individuals who have opted-in to anonymized research participation. From this, we identified 572 additional blood rapamycin samples from 316 individuals (87 females, 229 males) that contained sufficient information for further evaluation in the context of rapamycin bioavailability and dynamics over time after a single dose. As this evaluation was conducted retrospectively in a real-world cohort, a substantial proportion of the individuals were taking 6-mg commercial rapamycin (252 individuals (79.7%) and 473 timepoints (82.7%)), and only 10-mg dose was represented for the compounded formulation (Fig. 3a, Supplementary Table S6). Additionally, times between rapamycin dose administration and blood testing spanned 7 days, and intervals between blood tests for the same individual were generally 3 months or more.

We first evaluated whether blood rapamycin levels remained similar across doses and formulations within the larger cohort. To standardize evaluations, and allow for more reasonable comparison to the smaller study cohort, we restricted analysis to blood values obtained within 48 h after reported dose administration. This resulted in 167 datapoints, of which 84.4% were from the 6-mg commercial formulation group. Consistent with previous findings, average blood rapamycin level for each dose revealed similar patterns of response distribution (Fig. 3b), with variability in individual dose response that was not explained by factors such as gender or other medications, including Metformin, LDN, Acarbose, or NAD (Supplementary Table S8). Interestingly, the trends of increasing bioavailability by dose of commercial rapamycin observed in the small trial study (Fig. 1b) were not as apparent in this less tightly controlled cohort, even when all dose-to-test time ranges were included (Supplementary Figure S2a). To determine if this reflected any differences in the comparative bioavailability of each formulation in this larger cohort, we again averaged the blood rapamycin levels per mg of rapamycin taken for each dose. The resulting estimated bioavailability for compounded relative to commercial rapamycin was 30.26%, notably similar to the 31.03% estimate from the smaller cohort.

While uneven distribution of participants across doses and the single dosage (10 mg) of compounded rapamycin represented in this cohort limited reliable further exploration of dose linearity, the availability of multiple post-dose blood draw timepoints and longitudinal nature of this dataset permitted a preliminary investigation of the dynamics of rapamycin blood levels over time. We first explored the variation in blood rapamycin levels over the course of 1 week post-dose administration in participants using 6 mg of rapamycin (given the abundance of this dose in our dataset) who self-reported that they obtained blood rapamycin level evaluations from 1 to 7 days after taking their rapamycin dose (Supplementary Table S7). Averaging blood rapamycin levels for each day post dosing suggests that rapamycin blood levels peak 2 days after dosing, and decline gradually thereafter (Fig. 3c). These results were consistent when looking across all doses (Supplementary Figure S2b), as well as when subsetting for the most recent dose or doses at other timepoints (for patients with multiple dose-test timepoints within the dataset).

We next explored whether blood rapamycin levels were consistent over longer periods of time, as a robust number of individuals in this cohort (*n* = 228) had more than one dose-test timepoint over intervals of > 90 days. When evaluating all individuals with two timepoints, we found a significant increase in rapamycin levels from the first to second tests for the entire cohort (time 1 mean = 1.345, time 2 mean = 3.243, *t*(227) = 7.105, *p* < 0.001), though it should be noted that this was an overall effect, as not all individuals had increases. This effect remained when limiting to individuals who had two timepoints with a dose-test interval of 1–3 days (*n* = 56, time 1 mean = 2.093, time 2 mean = 3.793, *t*(55) = 2.668, *p* = 0.005; Fig. 3d), as well as by sex (female *n* = 11, time 1 mean = 0.818, time 2 mean = 2.773, *t*(10) = 2.288, *p* = 0.045; male *n* = 45, time 1 mean = 2.404, time 2 mean = 4.042, *t*(44) = 2.131, *p* = 0.039; Fig. 3e). Extending this analysis further to explore all datapoints over the full 1.5 year span of available datapoints (with a maximum individual timespan of 492 days) using linear regression analysis of blood rapamycin levels over time produced similar results of increasing blood rapamycin values over time (*F*(1, 580) = 33.735, *p* < 0.001, β = 0.234, *t* = 5.808, *p* < 0.001), which remained consistent after restricting to the most recent datapoints from patients with two or more samples (*F*(1, 314) = 14.648, *p* < 0.001, β = 0.211, *t* = 3.827, *p* < 0.001, Fig. 3f). However, to reduce potential bias of low values from new rapamycin users, we restricted the analysis to individuals who had been on rapamycin for more than 6 months. This resulted in a non-significant relationship between blood rapamycin values and time (*F*(1, 73) = 0.838, *p* < 0.363, β = −0.017, *t* = −0.915, *p* < 0.363), however, this appears to stem from a stabilization of rapamycin levels in this period for most individuals (Fig. 3g), though further studies will be required to elucidate this more conclusively.

## Discussion

As the geroscience field begins to validate geroprotective agents for their efficacy in improving healthy longevity, rapamycin has garnered particular interest [14]. As little is known about the bioavailability of different low dosages and formulations of rapamycin in humans, especially in the context of healthy aging, the current study aimed to establish a baseline reference framework for the relative bioavailability and blood level dynamics of both commercial and compounded rapamycin by measuring blood rapamycin levels in normative aging cohorts over time to better understand clinical impacts of such in our current and future work. We observed that both commercial and compounded rapamycin are absorbed and bioavailable, though compounded rapamycin has significantly less bioavailability per milligram dosed. We further observed that rapamycin levels may peak 2 days after dosing, and decrease thereafter, though individuals taking the same dose vary substantially in their specific blood rapamycin levels. Despite this, individuals measured at two subsequent timepoints appear to most commonly have the same or increasing blood rapamycin levels, with higher magnitude increases observed over 3 months than 2–4 weeks, and similar levels over periods longer than 6 months thereafter. While further studies with more granular levels of control (e.g., greater power, detailed dietary and metabolic monitoring, and others) will be required to fully establish the bioavailability and blood level dynamics of rapamycin at longevity doses, this work provides support for commonly utilized dosing strategies (primarily 6 mg/week) and suggests a baseline framework that can inform the design of future work on this topic.

Findings of differences in bioavailability of rapamycin between individuals were not altogether unexpected; however, lack of an association between differences in bioavailability due to baseline formulation potency differences, or sex, weight, BMI, length of time taking rapamycin, self-reported activity level, pre-existing health conditions, or other medications taken simultaneously, was surprising. As this result was consistent in both cohorts we studied, it is likely that dietary, metabolic factors, microbial interactions, plasma protein binding, individual genetics, underlying physiology, and potentially other lifestyle factors are contributing to differences in rapamycin bioavailability [36, 39]. For example, high-fat meals, grapefruit juice, and certain drugs (e.g., ketoconazole) have been shown to impact rapamycin’s bioavailability by up to 350% in some instances [37, 38]. Grapefruit juice in particular was shown to cause a longer peak and higher sustained levels, which may impact adverse events, while curcumin has been shown to substantially decrease bioavailability (> 75%) in animal models [38, 41]. While those data points were not available in the current investigation, additional work is ongoing to more fully explore these possibilities and should be considered for other future rapamycin bioavailability studies.

The differences in bioavailability between compounded and commercial formulations per milligram of rapamycin administered were substantial (31.03% bioavailability for compounded relative to commercial rapamycin). However, our collective results suggest that this relative bioavailability may be fairly consistent, and in the smaller and more carefully controlled portion of our study, we observed linearity of the dose–response relationship for both formulations. This provides needed evidence that compounded rapamycin is indeed bioavailable, and that similar blood rapamycin levels can be obtained with each given appropriate dosing and response monitoring. This is an important finding given the valid, widespread concern from experts on these points. Taken together, this may allow rapamycin users to more confidently benefit from the distinct advantages in cost, accessibility, dosing precision for reduction of AEs, and ease of placebo creation that compounded rapamycin provides.

It is important to note that we observed substantial inter-individual variability in blood rapamycin level concentrations at each dose of compounded and commercial rapamycin administered. This suggests differences in bioavailability between people taking the same rapamycin dose independent of formulation. While further investigation as to the underlying source of this variation is ongoing, until this effect is better understood individuals using longevity-doses of rapamycin would likely benefit from routine monitoring of blood rapamycin levels and personalized tailoring of individual dosing protocols.

Findings from our research database participant cohort that the highest blood rapamycin levels were observed 2 days after self-administration is novel data that provides reference points for future studies to more deeply explore the pharmacokinetics of longevity-doses of rapamycin. In combination with evidence from this cohort that weekly longevity doses of rapamycin maintain relatively stable bioavailability levels when taken consistently for 6 months or more, these findings provide important data supporting commonly utilized dosing strategies for longevity and may begin to address many of the questions related to blood rapamycin dynamics in normative aging individuals. However, it is important to note that this cohort was dramatically dominated by individuals taking 6 mg of rapamycin (likely due to the popularity of this dose within longevity medicine), which may limit the ability of findings to extend precisely to all other doses. Nonetheless, given the popularity of this rapamycin dose, we expect that these findings will bring value to the longevity field.

Despite the encouraging findings from the current study, it only just begins to explore the dynamics of longevity doses of rapamycin. More clearly elucidating how long and at what level rapamycin should be present in the blood for longevity-promoting effects remains necessary, and likely will only fully be addressed as longitudinal studies on functional outcomes in conjunction with markers of clinical effectiveness for healthy longevity increase [25]. In the intervening period, measuring Cmax (peak concentrations) of rapamycin may be a better indicator of bioavailability, as it reflects the total amount of rapamycin in the blood before clearance begins [42, 43]. Conversely, several PK studies of rapamycin suggest trough concentrations correlate with its efficacy and safety, as it has a long tail and thus represents the largest exposure time of organs to rapamycin [44, 45]. While it was beyond the scope of the current study to explore either of these measures further, future studies measuring blood rapamycin levels at peak, trough, and intermediate time points are undoubtedly required to more fully understand the best time points at which to measure rapamycin concentrations to correlate with clinical outcomes for healthy aging.

In conclusion, this study represents the largest investigation to date of real-world evidence on the relative bioavailability and blood level dynamics of longevity doses of commercial and compounded rapamycin in a healthy, normative aging cohort. Our findings suggest that therapeutic blood concentrations can be obtained from both formulations, however, the commercial formulation demonstrated approximately 3 × more potency per milligram than compounded. Regardless, overall trends suggest that rapamycin levels may peak 2 days after dosing and decrease in days thereafter, increase over the first few months of dosing, but then stabilize over longer periods of time. Our data revealed notable variability in individual blood rapamycin levels in response to the same dose of rapamycin, regardless of formulation, that was unexplained by sex, BMI, pre-existing health conditions, or other medications taken simultaneously. This variability suggests that routine measurement of blood rapamycin levels at standardized time points and dose personalization are likely the most reliable strategies for optimizing longevity dosing until rapamycin response is better understood. Taken together, our findings on real-world blood rapamycin data provide insights that pave the way for more comprehensive clinical evaluations of rapamycin’s blood availability dynamics in the future. Such studies will permit a more precise understanding of the association between rapamycin blood dynamics and clinical effectiveness metrics for healthy aging in normative aging individuals taking longevity-doses of rapamycin.

## Supplementary Information

Below is the link to the electronic supplementary material.

Supplementary file1 (PDF 999 KB)

## Data Availability

De-identified data is available upon request to the authors.
