# Supplementary material for: The bioavailability and blood levels of low-dose rapamycin for longevity in real-world cohorts of normative aging individuals
Source: GeroScience. 2025 Jan 28;47(4):5681–94. doi: 10.1007/s11357-025-01532-w (PMC12397450; doi:10.1007/s11357-025-01532-w)
Supplement: Supplementary file 1 — Supplementary file1 (PDF 999 KB) [file 11357_2025_1532_MOESM1_ESM.pdf]

Supplementary Figure S1

Click here to access/download;colour figure;Fig S1.tif

Fig S1

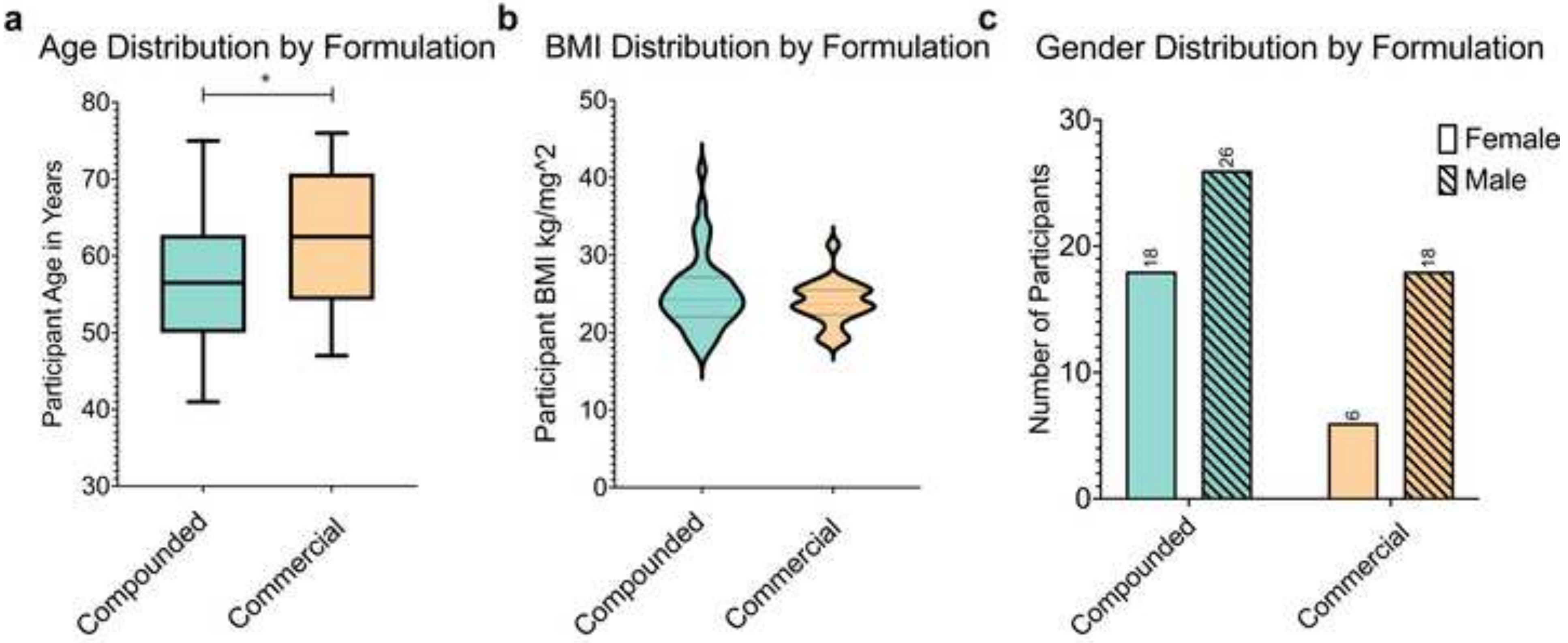

*p < 0.05

Supplementary Fig S2

a

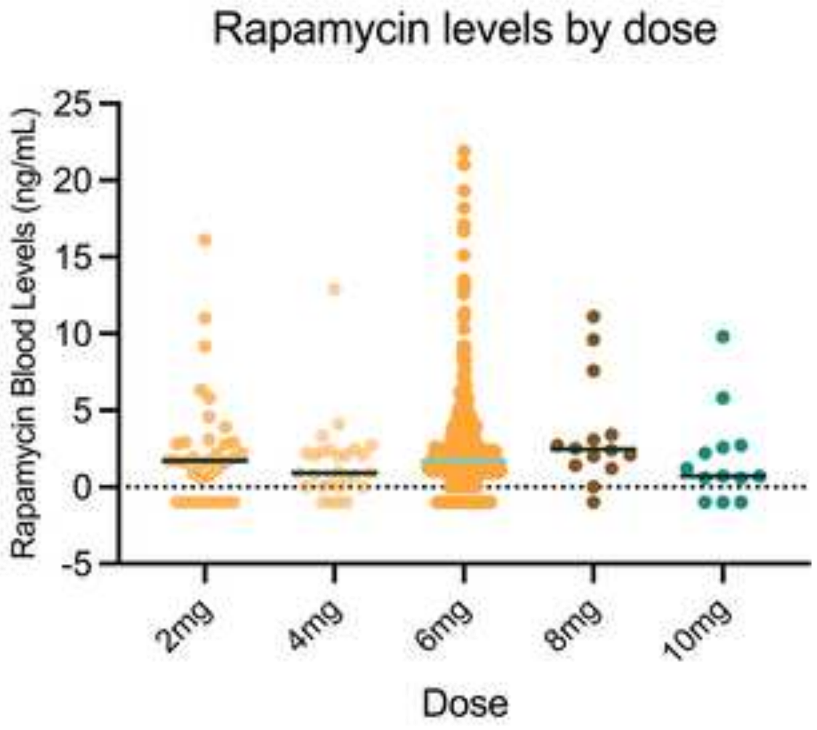

b

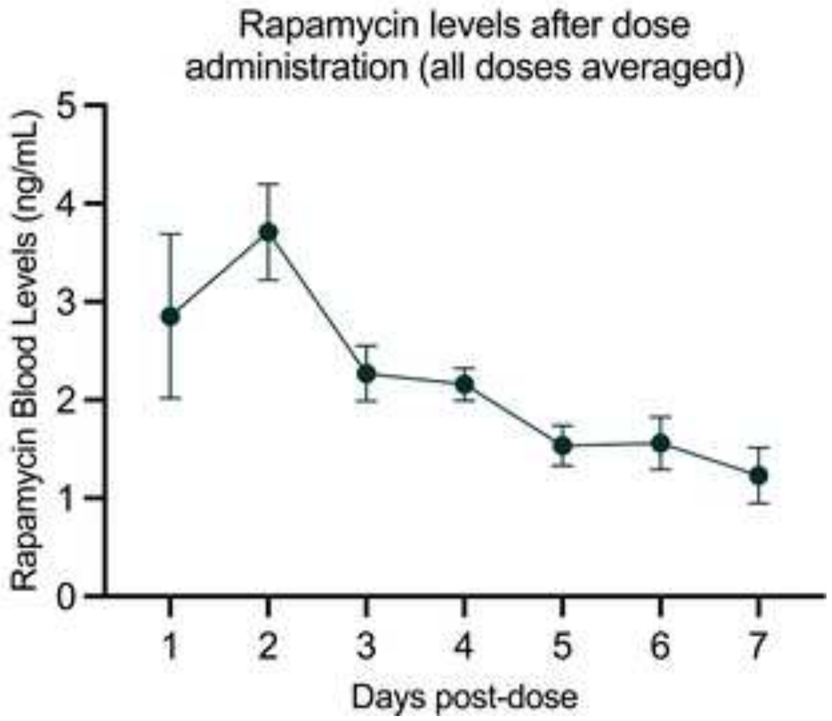

Supplementary Table S1: Comparative Demographic Distributions Across Formulation Types					
	Total	Compounded rapamycin	Commercial rapamycin	t(df) or X2 (df)	p-value
Age in years, mean (SD)	58.6 (9.5)	61.7 (9.1)	56.8 (9.3)	-2.05 (65)	0.044
BMI (kg/m2), mean (SD)	24.7 (4.3)	23.8 (2.9)	25.1 (4.8)	1.158 (65)	0.251
Sex, n female (%)	24 (35.8)	6 (26)	18 (41)		
Sex, n male (%)	43 (64.2)	17 (74)	26 (59)	2 (1)= 1.443	0.23

BMI: body mass index; SD: standard deviation

Supplementary Table S2: Dosage and formulation distribution by participant in a trial cohort

	Total	Female	Male
Dose, n (%)			
2 mg	19 (28.4)	10 (41.7)	9 (20.9)
4 mg	14 (20.9)	6 (25)	8 (18.6)
5 mg	1 (1.5)	-	1 (2.3)
6 mg	10 (14.9)	2 (8.3)	8 (18.6)
8 mg	1 (1.5)	-	1 (2.3)
10 mg	11 (16.4)	4 (16.7)	7 (16.3)
15 mg	11 (16.4)	2 (8.3)	9 (20.9)

Supplementary Table S3. Blood rapamycin evaluation at 24 hours post dosing for multiple factors

**Category**	***t, F****	***df***	***p*-value**
Metformin Use	1.513	65	0.135
Previous rapamycin use	0.497	12.09	0.628
Other Medications	1.794	20.94	0.087
Pre-existing conditions	1.643	65	0.105
Activity Level	1.162*	3, 65	0.331
BMI	0.428*	1, 65	0.515
Gender	1.197	60.48	0.236

t tests performed as Student's or Welch's as appropriate. F from ANOVA or regression analyses, as appropriate

**Supplementary Table S4.1. Effect of dosage on blood levels of rapamycin**								
**Group Protocol**	**Parameter**	**Estimate**	**Std. Error**	**df**	**t**	***p*-value**	**95% CI Lower**	**95% CI Upper**
1 Compounded	Intercept	-0.22	3.388	16.403	-0.065	0.949	-7.389	6.949
	**Dosage**	**0.173**	**0.071**	**25.584**	**2.442**	**0.022**	**0.027**	**0.318**
	BMI	0.005	0.106	18.456	0.045	0.964	-0.217	0.227
	Sex=1	-0.051	0.646	17.095	-0.079	0.938	-1.415	1.312
	Sex=2	-	-	-	-	-	-	-
	Age	0.015	0.029	17.207	0.537	0.598	-0.045	0.076
2 Commercial	Intercept	-4.22	1.984	42.269	-2.127	0.039	-8.223	-0.217
	**Dosage**	**0.697**	**0.111**	**38.548**	**6.269**	**<0.001**	**0.472**	**0.922**
	BMI	0.109	0.047	43.846	2.293	0.027	0.013	0.204
	Sex=1	0.335	0.459	41.576	0.732	0.469	-0.59	1.261
	Sex=2	-	-	-	-	-	-	-
	Age	0.033	0.02	39.213	1.659	0.105	-0.007	0.072

**Supplementary Table S4.2. Interaction of dosage by formulation**							
**Parameter**	**Estimate**	**Std. Error**	**df**	**t**	***p*-value**	**95% CI Lower**	**95% CI Upper**
Intercept	-3.173	1.73	65.91	-1.834	0.071	-6.627	0.282
Dosage	0.687	0.111	65.139	6.169	<0.001	0.464	0.909
Group=1	0.401	0.903	75.224	0.444	0.658	-1.398	2.201
Group=2	-	-	-	-	-	-	-
BMI	0.086	0.043	72.099	1.983	0.051	0	0.173
Sex=1	0.183	0.37	61.617	0.496	0.621	-0.555	0.922
Sex=2	-	-	-	-	-	-	-
Age	0.026	0.016	59.321	1.624	0.11	-0.006	0.058
**Group=1 * Dosage**	**-0.524**	**0.13**	**78.628**	**-4.026**	**<0.001**	**-0.783**	**-0.265**
Group=2 * Dosage	-	-	-	-	-	-	-

Supplementary Table S5: Overview of participants and number of measurements

	Total n	Compounded n	Commercial n
**Number of participants**	67	23	44
Participants with two measurements	21	15	6
Measurements with different doses	13	12	1
Measurements with the same dose	8	3	5

Supplementary Table S6: Comparative demographic distributions in a real-world user cohort						
	**Total Participants**			**Participants with two datapoints**		
	**Total**	**Female**	**Male**	**Total**	**Female**	**Male**
Dose, n (%)						
2 mg	20 (6.3)	7 (8)	13 (5.7)	14 (6.1)	4 (7)	10 (5.8)
4 mg	17 (5.4)	7 (8)	10 (4.4)	10 (4.4)	3 (5.3)	7 (4.1)
6 mg	252 (79.7)	66 (75.9)	186 (81.2)	191 (83.8)	45 (78.9)	146 (85.4)
8 mg	8 (2.5)	2 (2.3)	6 (2.6)	6 (2.6)	2 (3.5)	4 (2.3)
10 mg	12 (3.8)	3 (3.4)	9 (3.9)	4 (1.8)	1 (1.8)	3 (1.8)

Supplementary Table S7. Distribution of duration in days between rapamycin dose and blood testing

Days	**Total n**	**Female n**	**Male n**
1	74	23	51
2	94	18	76
3	87	17	70
4	93	23	70
5	86	18	68
6	76	27	49
7	72	24	48

Supplementary Table S8. Effects of medication use on rapamycin blood levels in the Observational Research Database cohort

Category	*t*	*df*	*p*-value
Metformin	0.531	371.9	0.596
LDN	0.624	102.148	0.534
Acarbose	0.229	50.246	0.82
NAD	11.244	45.936	0.22
Gender	1.678	68.951	0.098

Note: analyses performed for all participants taking 6mg of rapamycin for blood measurements obtained within 2 days of dose administration. t tests were performed for individuals using the medication at the time of testing vs those not using the medication at the time of testing.
